# Green synthesis of zinc oxide nanoparticles (ZnO-NPs) by *Streptomyces baarnensis* and its active metabolite (Ka): a promising combination against multidrug-resistant ESKAPE pathogens and cytotoxicity

**DOI:** 10.1186/s12866-024-03392-4

**Published:** 2024-07-09

**Authors:** Mohamed H. Kalaba, Gamal M. El-Sherbiny, Emad A. Ewais, Osama M. Darwesh, Saad A. Moghannem

**Affiliations:** 1 Botany and Microbiology Department, Faculty of Science (Boys), Al-Azhar University, Nasr City, Cairo, 11884 Egypt; 2 Agricultural Microbiology Department, National Research Centre, Dokki, Cairo Egypt

**Keywords:** ZnO-NPs, Biosynthesis, Nanoemulsion, Characterization, Antibacterial activity, Cytotoxicity

## Abstract

Various eco-friendly techniques are being researched for synthesizing ZnO-NPs, known for their bioactivity. This study aimed at biosynthesizing ZnO-NPs using *Streptomyces baarnensis* MH-133, characterizing their physicochemical properties, investigating antibacterial activity, and enhancement of their efficacy by combining them with a water-insoluble active compound (Ka) in a nanoemulsion form. Ka is a pure compound of 9-Ethyl-1,4,6,9,10-pentahydroxy-7,8,9,10-tetrahydrotetracene-5,12-dione obtained previously from our strain of *Streptomyces baarnensis* MH-133. Biosynthesized ZnO-NPs employing *Streptomyces baarnensis* MH-133 filtrate and zinc sulfate (ZnSO_4_.7H_2_O) as a precursor were purified and characterized by physicochemical investigation. High-resolution-transmission electron microscopy (HR-TEM) verified the effective biosynthesis of ZnO-NPs (size < 12 nm), whereas dynamic light scattering (DLS) analysis showed an average size of 17.5 nm. X-ray diffraction (XRD) exhibited characteristic diffraction patterns that confirmed crystalline structure. ZnO-NPs efficiently inhibited both Gram-positive and Gram-negative bacteria (MICs: 31.25–125 µg/ml). The pure compound (Ka) was combined with ZnO-NPs to improve effectiveness and reduce dose using checkerboard microdilution. Niteen treatments of Ka and ZnO-NPs combinations obtained by checkerboard matrix inhibited *Klebsiella pneumonia*. Eleven combinations had fractional inhibitory concentration index (FICi) between 1.03 and 2, meaning indifferent, another five combinations resulted from additive FICi (0.625–1) and only one combination with FICi of 0.5, indicating synergy. In the case of methicillin-resistant *S. aureus* (MRSA), Ka-ZnO-NPs combinations yielded 23 treatments with varying degrees of interaction. The results showed eleven treatments with indifferent interaction, eight additive interactions, and two synergies with FICi of 0.5 and 0.375. The combinations that exhibited synergy action were transformed into a nanoemulsion form to improve their solubility and bioavailability. The HR-TEM analysis of the nanoemulsion revealed spherical oil particles with a granulated core smaller than 200 nm and no signs of aggregation. Effective dispersion was confirmed by DLS analysis which indicated that Ka-ZnO-NPs nanoemulsion droplets have an average size of 53.1 nm and a polydispersity index (PI) of 0.523. The killing kinetic assay assessed the viability of methicillin-resistant *Staphylococcus aureus (*MRSA) and *K. pneumonia* post-treatment with Ka-ZnO-NPs combinations either in non-formulated or nanoemulsion form. Results showed Ka-ZnO-NPs combinations show concentration and time-dependent manner, with higher efficacy in nanoemulsion form. The findings indicated that Ka-ZnO-NPs without formulation at MIC values killed *K. pneumonia* after 24 h but not MRSA. Our nanoemulsion loaded with the previously mentioned combinations at MIC value showed bactericidal effect at MIC concentration of Ka-ZnO-NPs combination after 12 and 18 h of incubation against MRSA and *K. pneumonia*, respectively, compared to free combinations. At half MIC value, nanoemulsion increased the activity of the combinations to cause a bacteriostatic effect on MRSA and *K. pneumonia* after 24 h of incubation. The free combination showed a bacteriostatic impact for 6 h before the bacteria regrew to increase log_10_ colony forming unit (CFU)/ml over the initial level. Similarly, the cytotoxicity study revealed that the combination in nanoemulsion form decreased the cytotoxicity against kidney epithelial cells of the African green monkey (VERO) cell line. The IC_50_ for Ka-ZnO-NPs non-formulated treatment was 8.17/1.69 (µg/µg)/ml, but in nano-emulsion, it was 22.94 + 4.77 (µg/µg)/mL. In conclusion, efficient Ka-ZnO-NPs nanoemulsion may be a promising solution for the fighting of ESKAPE pathogenic bacteria according to antibacterial activity and low toxicity.

## Introduction

Presently, there is a significant amount of attention focused on the field of nanotechnology, which may be classified into three distinct categories: physical, chemical, and biological [1, 2]. While all three methods have been used for nanoparticle synthesis, both the chemical and physical processes are associated with expenses related to equipment, potential environmental damage, and the need for high pressures and temperatures [3]. On the contrary, the green production of nanoparticles is increasingly being carried out via biological materials. They are preferable to alternative methods in several ways, including being low-cost, eco-friendly, and typically having one-step protocols [4–6]. The assembly of NPs with biological agents, including plant extracts, microbial extracts, and metabolites, can be employed to generate eco-friendly structures without the necessity of evolving manufacturing processes, such as pressures and temperatures, which generate deleterious compounds [7, 8]. A variety of bio-systems, most notably bacteria, actinobacteria, fungi, and plants have been able to safely make metallic nanoparticles [1, 4, 9, 10]. The most significant group of microorganisms that could be utilized to develop new pharmaceutical and commercial products, including antibacterial medications, are bacteria, especially actinobacteria [11, 12]. *Actinomycetes*, of which *Streptomyces* sp. is a part, are well able to produce potent bioactive compounds like antibiotics. They are also considered appropriate nanoparticle fabricators since a variety of actions can be used to create them both extracellularly and intracellularly [13, 14].

In recent decades, the global production and utilization of nanoscale materials, including metal and metal oxide-based nanoparticles, carbon nanoparticles, organic nanoparticles, and quantum dots, have significantly increased. Among these, metal and metal oxide nanoparticles are widely employed in biomedicine and environmental applications due to their unique optical, chemical, mechanical, electrical, and magnetic properties [15, 16]. Zinc is a fundamental element for humans, animals, and microorganisms. Zinc maintains important physiological functions including oxidative stress, DNA repair, DNA replication, and cell cycle progression [17]. Due to their distinctive characteristics, ZnO-NPs have received a lot of interest. Compared to their usual Zn sources, they have a higher rate of absorption, reduced toxicity, and improved biocompatibility and bioavailability [18]. ZnO-NPs have been found to have a wide range of biological and therapeutic properties, including antibacterial, antiprotozoal, antioxidant, and anticancer properties [19, 20]. The antibacterial effect of ZnO-NPs has been explained by different mechanisms [21]. ZnO-NPs’ surface polarity and positively charged surface may have an impact on how well they fight germs. Zn^+ 2^ NP ions, which are positively charged, interact with negatively charged bacteria, causing the bacteria to get firmly attached to the ions and eventually die [22, 23].

Biologists are becoming more interested in using nanometals, namely ZnO-NPs, for a range of applications including gene transport, medication administration, material detection in biological samples, and combating diseases and cancers. This is due to the unique properties of nanometals [24, 25]. Nanometals may synergistically boost the efficiency of standard antibacterial agents, such as antibiotics or natural compounds to combat microbial diseases [26]. This synergy results from a variety of processes, including enhanced penetration into bacterial cells, breakdown of cell membranes, and the generation of reactive oxygen species. This technique, which takes use of the complementary features of nanometals and antibacterial compounds, provides a powerful and diverse alternative for combating antibiotic-resistant microbes and decreasing the burden of infectious illnesses [27]. Nanoemulsions are an adaptable approach for increasing the bioavailability of water-insoluble active substances, thereby boosting their medicinal efficiency. Nanoemulsions are colloidal dispersions made up of tiny droplets of oil suspended in an aqueous phase. This unique structure allows hydrophobic compounds to be encapsulated within the oil droplets, overcoming problems such as poor solubility and absorption. Nanoemulsions enhance the surface area of drug particles and allow for quick rapid dissolution upon administration by reducing them to nanometer size. Consequently, nanoemulsions provide better drug delivery efficiency, accurate dosage control, and improved therapeutic results, making them a potential formulation technique for a variety of pharmaceutical and biological applications [28, 29].

Herein, *Streptomyces baarnensis* MH-133 had been previously isolated, identified, and able to produce a water-insoluble pure compound named 9-Ethyl-1,4,6,9,10-pentahydroxy-7,8,9,10-tetrahydrotetracene-5,12-dione (Ka) that had shown activity against ESKAPE pathogenic bacteria [30, 31]. ESKAPE pathogens, a class of bacteria, are multidrug-resistant and present a significant hazard to human health. *Enterococcus faecalis, Staphylococcus aureus, Klebsiella pneumoniae, Acinetobacter baumannii, Pseudomonas aeruginosa, and Enterobacter* species comprise the acronym ESKAPE. So, this paper aimed to biosynthesize ZnO-NPs using the filtrate of this strain, formulate a nanoemulsion containing ZnO-NPs and water-insoluble Ka combination, and evaluate their effectiveness against some pathogenic bacteria and cytotoxicity.

## Materials and methods

### Microorganisms

The clinical bacterial isolates and standard strains used in this study as test microorganisms were obtained from the Bacteriology laboratory at Botany and Microbiology Department, Faculty of Science, Al-Azhar University. These microorganisms included Gram-negative (*Enterobacter cloacae, Acinetobacter baumanii, Escherichia coli, Klebsiella pneumonia, Pseudomonas aeruginosa*, and *Salmonella typhi-*ATCC-6539) and Gram-positive (*Enterococcus faecalis, Staphylococcus aureus, and Bacillus subtilis-ATCC-6633).* Our *Streptomyces baarnensis* MH-133 was used to synthesize ZnO-NPs previously isolated and identified [30].

### Extracellular fabrication of ZnO-NPs by *Streptomyces baarnensis* MH-133

Culture of *Streptomyces baarnensis* MH-133 was obtained by inoculating 50 ml of marine broth medium with 8% (v/v) of fresh culture in a 250 ml flask and incubating it on a rotary shaker at 150 rpm and 30 °C for 12 days. To separate the cell-free filtrate (CFF) from the pellet residue, the culture of *Streptomyces baarnensis* MH-133 was filtered through a cotton layer and centrifuged at 10,000 rpm for 10–15 min. The filtrate was introduced in a 1:1 (v/v) ratio to the reaction vessels containing zinc sulfate solution (1 gm/100 ml distilled water) (ZnSO_4_.7H_2_O, ADWIC, Egypt), and controls (only bacterial filtrate (the positive control) and metal solution without supernatant (the negative control)) were kept separate for each reaction [31]. The reaction mixture was incubated in a rotary shaker at 120 rpm for 72 h at 37 °C and initial pH 7 [32]. The color of the reaction mixture revealed the presence of biosynthesized metal nanoparticles. The greatest surface plasmon resonance peak of the reaction mixture was determined to confirm the findings. The ultraviolet-visible spectrum was analyzed at 200–800 nm using the Unico 2100 UV-visible Spectrophotometer USA [33].

### Purification of biosynthesized ZnO-NPs

The reaction mixture including ZnO-NPs was subjected to centrifugation at a speed of 10,000 rpm for 15 min. To completely remove any remaining molecules, the ZnO-NPs, which were in the form of precipitate, were subjected to three rounds of washing using sterile deionized water and re-centrifugation at 1000 rpm for 5 min. The metal nanoparticle solution was dried in a hot air oven at a temperature of 60 °C until it achieved a stable weight [34].

### Characterization of biosynthesized ZnO-NPs

Biosynthesized nanoparticles were characterized by measuring their UV-visible spectra (Unico 2100 UV-visible Spectrophotometer, USA), which ranged in wavelength from 200 to 800 nm [35].

Using a Fourier Transform Infrared Spectroscopy (FTIR) spectrophotometer in diffuse reflectance mode, the most likely functional groups of biomolecules responsible for the reduction and capping of ZnO-NPs were identified using a Fourier transform infrared spectrometer (Agilent System Cary 630 FTIR Model Chemical Department, Faculty of Science, Al-Azhar University, Cairo, Egypt), the samples were examined in the infrared range of 4000 –400 cm^− 1^. The obtained spectral data was compared to the reference chart to determine which functional groups were present in the sample [36]. The X-Ray diffraction pattern of ZnO-NPs was done at the National Center for Radiation Research and Technology (NCRRT) in Cairo, Egypt, using a Shimadzu apparatus with a nickel filter and a Cu-Ka target (Shimadzu Scientific Instruments (SSI), Kyoto, Japan). HR-TEM was used to determine the morphology of ZnO-NPs (JEOL 2100, Japan, at the National Research Center (NRC) in Giza, Egypt). ZnO-NPs suspension was drop-coated onto a carbon-coated copper grid, which was then inserted into a specimen holder after curing to visualize it. By using HR-TEM micrographs, the diameters and morphologies of ZnO-NPs were determined [1]. Dynamic Light Scattering (DLS) measurements were conducted with a Malvern Zetazier Instrument at the National Center for Radiation Research and Technology (NCRRT), Cairo, Egypt, to determine the particle size distribution of nanoparticles. The range of measurements was between 0.1 and 1000 m [37].

### Preparation of stable ZnO-NPs nanofluid

A nanofluid is a colloidal solution of solid nanoparticles distributed in a suitable fluid. It was prepared by dispersing the needed concentration of ZnO-NPs in glycerol as base fluid with continuous mixing using the Homogenizer Digital High Disperser Mixer (Heidolph, India). di-ammonium hydrogen citrate (Merck- Germany) was dissolved in distilled water. The weight ratio of nanoparticles to di-ammonium hydrogen citrate was kept at 1:1 (w/w), and the ratio of glycerol base to aqueous solution of dispersant was 60:40 (v/v). The two solutions were continuously mixed for 3 h. at 15,000 rpm at room temperature until gave a homogeneous stable suspension of ZnO-NPs [38].

### Antibacterial activity of nanofluid and water suspension of ZnO-NPs

The antibacterial activity was carried out on ZnO-NPs water suspension and nanofluid via the agar cup diffusion technique against *Klebsiella pneumonia* and *Staphylococcus aureus* as a model of Gram-negative and Gram-positive, respectively. Agar cups (7 mm diameters) were cut in plates containing Mueller-Hinton agar, surfacy spread with the test bacteria (optical density (OD) equivalent to MacFarland standard 0.5). Agar wells were divided into two groups: the first was loaded with 100 µl of nanoparticle nanofluid samples (5 mg/ml) of Zn nanoparticles separately, while the second group was loaded with 100 µl of nanoparticle suspension samples containing the same concentration as mentioned above of ZnO-NPs. As a control, agar wells with just a nanofluid base and no nanoparticles were employed. These plates were incubated at 37 °C for 24 h after being maintained at 4 °C for 2 h to allow for maximum diffusion. The test substances’ antibacterial potential was then found by measuring the diameter of the zone of inhibition [39].

### Minimal inhibitory concentration (MIC) of ZnO-NPs nanofluids

The MIC values of ZnO-NPs in the form of nanofluids against multidrug-resistant (MDR) strains, including *Acinetobacter baumanii, Escherichia coli, Enterobacter cloacae, Klebsiella pneumonia, Pseudomonas aeruginosa, Enterococcus feacalis*, and *Staphylococcus aureus*, in addition to *Bacillus subtilis* ATCC-6633 and *Salmonella typhi* ATCC-6539, were determined by broth microdilution assay. One hundred microliters of the tested samples were added to sterile microtiter plate wells filled with 100 µl of double-strength Mueller Hinton (MH) broth to obtain final concentrations of 2000, 1000, 500, 250, 125, 62.5, 31.25, 15.75, and 7.8 µg/ml of ZnO-NPs nanofluid. In all wells except the control well, 5% (V/V) bacterial cell suspension (OD corresponding to 0.5 McFarland standard) was added. Three sets of control wells were used; the first comprised sterile Mueller-Hinton broth to ensure sterility. The second control well was filled with MH broth and bacterial suspension to see if the broth was enough for bacterial growth. The third set of control wells was filled with MH broth and ZnO-NPs nanofluid concentrations were utilized to compare their color with the same tested concentrations (without bacteria) [40]. The plates were incubated at 37 °C for 24 h. Each well was treated with 30 µl of resazurin solution (HiMedia) containing 0.18% W/V to detect bacterial growth. The plate was then re-incubated for 4 h. An observation of a transition from blue to red, purple, or pink signified the existence of bacterial proliferation. In contrast, the absence of growth was indicated by the sterile control well’s lack of color change. The experiment was conducted in triplicate, and the means were determined [41].

### Evaluation of the Ka and ZnO-NPs combinations using checkerboard microdilution assay

The experiment was conducted using 96-well microplates filled with Mueller-Hinton Broth (Difco) with Ka concentrations of 300, 150, 75, 37.5, 18.75, 9.375 and zero µg/ml in the columns and ZnO-NPs nanofluid of 62.5, 31.25, 15.6, 7.8, 3.9, 1.95 and zero µg/ml along the rows, then combined on the plate in a checkerboard style. The inoculum size of 5% (V/V) of bacterial suspension (OD 0.5 McFarland standard) was distributed in each well. The checkboard microdilution method was performed in duplicate, and the bacterial growth was checked after 24 h of incubation at 37 °C [42]. The growth of MDR indicator strains *Klebsiella pneumonia* and *Staphylococcus aureus* that were used as a test strain in this assay was evaluated by the addition of 30 µl of resazurin solution (0.18%W/V) (HiMedia) to each well as described in the MIC experiment [43]. The interaction was assessed algebraically by determining the fractional inhibitory concentration index (FICi). FICi was calculated according to the equations below:

$$\rm\text{FICi}=\text{FIC}\,Ka\,+\text{FIC}\,\text{ZnO}-\text{NPs}$$

Where, FICi is the fractional inhibitory concentration index, FIC ZnO-NPs = MIC of ZnO-NPs in combination / MIC of ZnO-NPs when alone, and FIC Ka = MIC of Ka in combination / MIC of Ka when alone. Interpretation of the results was as follows: Partial synergy/addition: FICi > 0.5 ≤ 1.0; Synergism: FICi ≤ 0.5; Indifference: FICi > 1 ≤ 2.0 Antagonism [FICi] = mean > 2. Synergy is defined as the condition in which the total effect of a combination of elements is greater than the sum of the impacts of the individual components. Partial synergy and addition: The presence of an additive effect in a combination is determined by whether the combined effect is equivalent to the totality of the effects of the individual components. An effect of a combination is considered indifferent if its effects are equivalent to those of the most active component. Antagonism arises when the combined effect of two substances is lower than the effect of the most potent substance used alone [44].

### Development and characterization of nanoemulsion loaded with ka and ZnO-NPs combination

Both ka and ZnO-NPs were poorly or sparingly soluble in water, olive oil as the oil phase, S_mix_ is a mixture of tween 80 and glycerol as surfactant and co-surfactant respectively, and distilled water as aqueous phase were used to prepare nanoemulsion which will carry the components that show synergism in checkerboard assay. The nanoemulsion formula (vehicle) contains 45% S_mix_ at a ratio of 2:1, 7.5% of oil ratio, and 47.5% of water (w/w). Nanoemulsions containing Ka and ZnO-NPs were prepared by dissolving the appropriate concentration of Ka in olive oil at the weight ratio of the prepared formula. The desired concentration of ZnO-NPs was dissolved in the weight ratio of glycerol at the required quantity of S_mix_ (Tween 80 and glycerol) of the formula. The S_mix_ containing dissolved ZnO-NPs was added to the oil phase containing the desired concentration of Ka and mixed up with the help of a mechanical shear mixer at 8500 rpm for 30 min. The final formulation was brought to 100% w/w by gradually adding distilled while combining continuously at 12,000 rpm for 30 min. The prepared nanoemulsion formulations were observed for 24 to detect any turbidity, precipitation, creaming, cracking, or phase separation [45]. HR=TEM (JEOL 2100 Japan) at the National Research Centre, Cairo, Egypt, was employed as a means for morphological examination and globular-size (droplets) confirmation. Before the analysis, the sample was diluted in Millipore water (1:100), and a sample drop was placed on a copper grid to investigate it. The droplet size of the nanoemulsions was determined by photon correlation spectroscopy using a Zetasizer 1000 HS (Malvern Instruments, Worcestershire, UK). The nanoemulsion sample was diluted in Millipore water (1:100). Light scattering was monitored at 25 °C at a 90° angle. Average droplet size, polydispersity index, and size distribution of nanoemulsion were determined. 

### Comparative studies between formulated combinations (Nanoemulsion) and non-formulated combinations

Comparative studies included the determination of time-kill assay of Ka and ZnO-NPs combinations without and with nanoemulsion formulation against MDR *Klebsiella pneumonia* and MRSA and cytotoxicity against normal cell lines.

#### Time kill assay study

This study was performed according to CLSI, [46]. the kill measurement was determined by the actual reduction in viable counts at different time intervals for each tested strain. MDR indicator strains *Klebsiella pneumonia* and MRSA was chosen to perform a time-kill assay. Thus, 0.5 McFarland standard suspension of bacteria was prepared to have 50 ml of approximately ~ 10^6^ CFU/ml in nutrient broth. The formulated and non-formulated combinations were prepared and added to the corresponding culture at the half MIC and MIC values obtained from the checkerboard assay. The cultures were incubated at 37 °C. For estimation of cell viability at different intervals (0, 1, 2, 3, 4, 5, 6, 12, 18, and 24 h), one ml of each treatment as well as controls (positive control represent medium inoculated with tested bacteria, autogenic control represent medium plus empty nanoemulsion inoculated with tested bacteria) were subjected to serial dilutions using 0.85% sterile saline solution and 100 µl of each dilution was plated onto 90 mm in diameter Petri plates containing nutrient agar in triplicate and incubated for 18 h at 37 °C. after that, the CFU of each plate was calculated. The criteria for the classification of formulated and non-formulated combinations as bacteriostatic or bactericidal agents were assigned according to Lorian, [47]. Bacteriostatic was defined as a reduction of < 3 log CFU/mL and bactericidal effects were defined as a reduction of ≥ 3 log CFU/mL after 24 h of incubation, compared to the count of the initial inoculum.

#### Cytotoxicity study

A cytotoxicity assay of ka-ZnO-NPs in the form of formulated and non-formulated combinations in addition to an empty nanoemulsion formula was performed according to Kalaba et al. [48]. One hundred microliters of VERO cells (10^5^ cells/ml) were seeded in 96 well plates containing medium with serial concentrations started with the MIC value of the combination obtained from checkerboard assay in the form of formulated and non-formulated combinations. For 24 h, the cells were cultured at 37 °C, 5% CO_2_, 95% air, and 100% relative humidity. After incubation, the solution was withdrawn from the plate. A 100 µl aliquot of media with 1 mg/ml of 3-(4,5-dimethylthiazol-2-yl)-2, 5-diphenyl-tetrazolium bromide (MTT) was added to the plate. The cells were re-incubated for 4 h before the fluid was withdrawn. To dissolve the crystals, 100 µl of DMSO was added to the plate and shaken well. The optical density (OD) of the converted dye at 570 nm was measured in an ELISA reader to evaluate both the IC_50_ value and the viability of Vero cells according to this equation:

Cell viability (%) = ((OD treatment) / (OD control)) Χ100.

#### Observation of the effects nanoemulsion combinations on bacterial cells via TEM

To detect the effect of formulated combinations on bacterial cells, transmission electron microscopic observations were carried out on MRSA and *Klebsiella pneumoniae* as examples of Gram-positive bacteria and Gram-negative, respectively. Standards suspensions (0.5 McFarland) of tested bacteria were inoculated (5% V/V) in a 100 ml conical flask containing 20 ml nutrient broth medium. The nanoemulsion combination of Ka- ZnO-NPs at sublethal concentration (18.75/3.9 (µg/µg)/ml of ka- ZnO-NPs against *Klebsiella pneumoniae* and18.75/1.95 (µg/µg)/ml against MRSA, respectively) corresponding to each bacterial type was added to the prepared media. Also, flasks containing only media were prepared with the same volume (control)and inoculated with the prepared bacterial suspension. Both of controls and the treated cells were incubated at 37 °C on a rotary shaker at 120 rpm for 18 h [49]. After incubation, both control and treated cells were separately collected and subjected to the steps described by Foda et al. [43], to prepare the ultrathin sections. These sections were then investigated at 80 KV using (JEOL 1010) Transmission Electron Microscope at The Regional Center for Mycology and Biotechnology (RCMB), Al-Azhar University, Cairo, Egypt.

### Statistical analysis

Data were expressed as the mean ± standard error of the mean (std.er.). To determine differences between factors’ levels, a pairwise multiple comparison was performed using the Holm-Sidak test using minitab18 software extended with a statistical package, and Microsoft™ Excel^®^ 2016 was used to statistically analyze the data. statistical significance was determined when *p* < 0.05.

## Results and discussion

### Extracellular biofabrication and characterization of biosynthesized ZnO-NPs

**Fig. 1:**
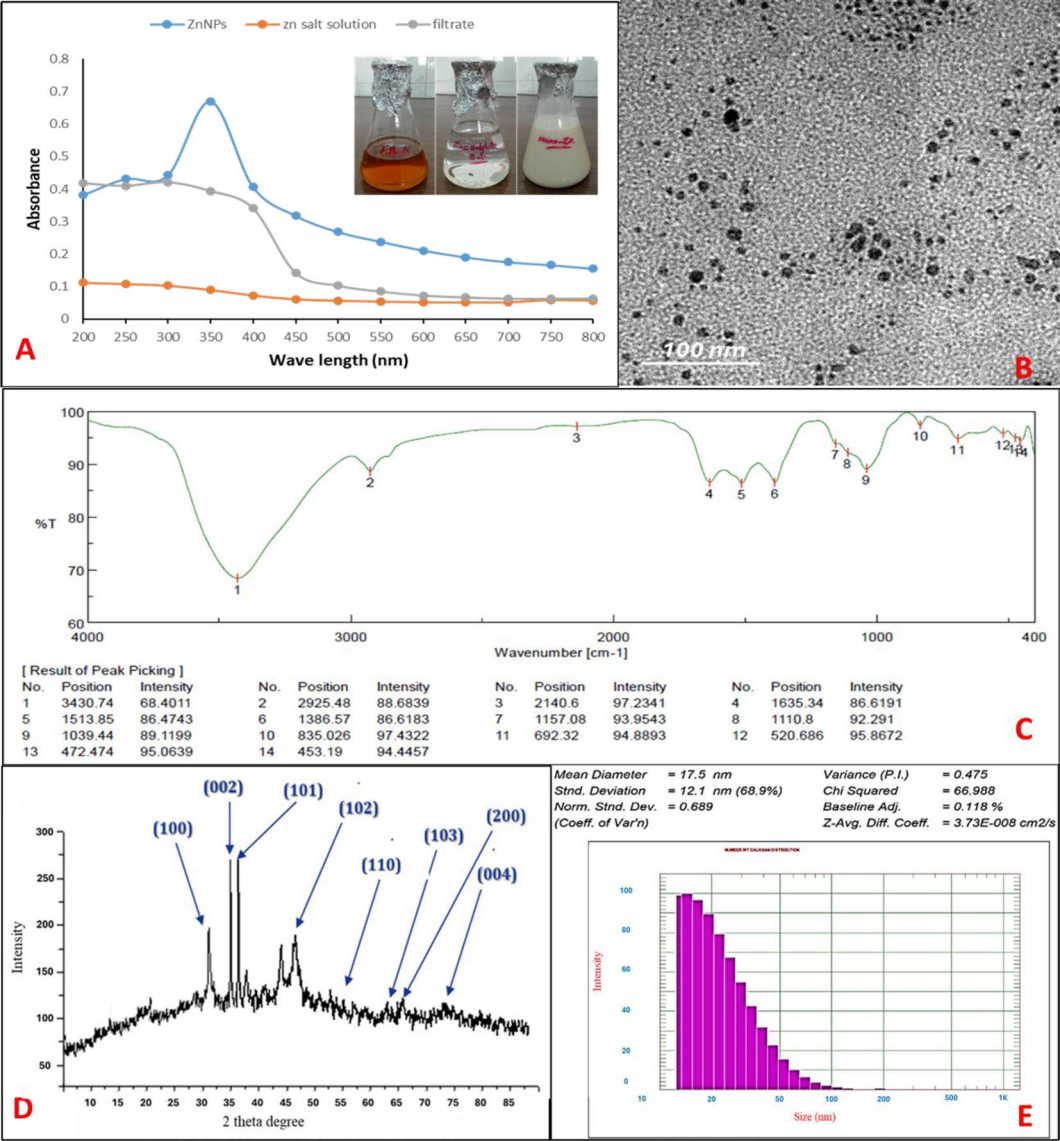
Biosynthesis and characterization of ZnO-NPs; (**A**) Visual observation of ZnO-NPs formation and UV; (**B**) TEM (**C**) FTIR; (**D**) XRD; (**E**): DLS

The extracellular biosynthesis of ZnO-NPs was prepared through the addition of a cell-free filter of *Streptomyces baarnensis* MH-133 to an equal volume of zinc precursor (1:1 v/v). The primary confirmation of ZnO-NPs biosynthesis was done by the changes in the color of the ZnSO_4_.7H_2_O solution from colorless to yellowish-white or turbid white after the addition of the cell-free filtrate (CFF) aseptically in equal volumes as shown in Fig. 1A. To generate metal nanoparticles, CFF of *Streptomyces* species has been used in several previous studies [50]. The main reductants for converting zinc salt to zinc oxide nanoparticles are the proteins and amino acids found in the CFF of *Streptomyces rochei*, and they can be employed to synthesize metal nanoparticles [51]. Because the generation of color is based on the excitation of surface plasmon resonance (SPR) in metal, the findings were confirmed by estimating the maximal SPR peak of the reaction mixture using UV-visible spectrum analysis. Because peak positions and shape are sensitive to particle size, UV-visible absorbance spectroscopy is a particularly helpful tool for analyzing metal nanoparticles. The SPR peak of ZnO-NPs generated in the reaction combination of *Streptomyces baarnensis* MH-133 filtrate and ZnSO_4_.7H_2_O solution was at 350 nm. The two types of controls, bacterial filtrate and metal solution do not exhibit any sharp peaks like those obtained from metal nanoparticles, as shown in Fig. 1A. This result was in line with findings published by Yusof and colleagues [52], who biosynthesized ZnO-NPs using CFF and bacterial cells from *Lactobacillus plantarum* TA4. They observed that the UV-vis absorption spectra maxima were at 349 and 351 nm, respectively. Ehsan and Sajjad [53] used zinc sulfate as a precursor and showed that the fabrication of ZnO-NPs using *Ficus carica* leaf extract demonstrates the highest peak absorption at 360. TEM image of the biosynthesized ZnO-NPs with the CFF of *Streptomyces baarnensis* MH-133 explain that ZnO-NPs are spherical-shaped with particle sizes less than 12 nm in a water-suspended sample with some aggregations as shown in Fig. 1B. Santhoshkumar et al. [54] reported that the different magnification levels of the SEM pictures clearly showed the presence of spherical ZnO-NPs with an average diameter of 70 nm.

The FTIR analysis was performed to gather information regarding the functional groups existing within the synthesized ZnO-NPs. The aim was to gain insights into the transformation process of these nanoparticles, transitioning from basic inorganic zinc salts (ZnSO_4_.7H_2_O) to elemental zinc. This transformation is influenced by a variety of metabolites that serve as agents for reducing, and capping. In Fig. 1C, the FTIR spectrum of ZnO-NPs displays prominent absorption peaks at 3413, 2925, 1633, 1415, 1392, 1099, 1041, 873, 769, and 603 cm^− 1^. The broad and intense absorption peak at 3413 cm^− 1^ is typically associated with the presence of hydroxyl functional groups, while the peak at 2925 cm^− 1^ corresponds to the stretching of C-H bonds found in methylene groups, typically present in proteins. The peaks at 1633 and 1415 cm^− 1^ are indicative of the binding vibrations related to the amide I band of proteins, involving N-H stretching. The peak at 1392 cm-1 suggests the presence of organic molecules or functional groups associated with specific chemical bonds. It could be related to C-H bending vibrations in aliphatic compounds. The peak at 1099 cm-1 typically corresponds to the stretching vibrations of C-O bonds, which can be found in various organic compounds, including alcohols, esters, or carboxylic acids. It may indicate the presence of such groups on the surface of the ZnO-NPs. The peak at 1041 cm-1 can be associated with C-H bending vibrations in organic compounds, possibly indicating the presence of hydrocarbons or aliphatic groups while the peak at 873 cm-1 may be linked to C-H bending vibrations. The peak of 603 cm-1in this region often indicates the presence of metal-oxygen (M-O) bonds. In the case of ZnO-NPs, this peak could be related to Zn-O vibrations, which are characteristic of zinc oxide. According to Kapoor et al. [55] proteins, primarily through reducing enzymes located on the cell membrane of microorganisms or released into the growth medium as extracellular enzymes, can play a role in the biosynthesis of nanoparticles. Under certain conditions, specific organic functional groups on the microbial cell wall are necessary for non-enzymatic synthesis as they enhance the reducibility of metal ions. Our study has detected the presence of amide I and II bands and proteins, which may contribute to the synthesis and capping of the metals [56]. The large peak at 3500 cm^− 1^ is associated with the surface absorption of molecular water and CO_2_ by the nanoparticles [57]. The fingerprint region of the ZnO-NPs, characterized by absorption peaks below 700 cm^− 1^, resembles that of metal oxides. The extracellular filtrate of *S. cerevisiae* contains various functional groups such as proteins, alcohols, phenolic groups, fatty acids, and carbohydrates. The extracellular proteins can prevent nanoparticle aggregation and enhance their stability by forming a coating [58].

In the XRD spectrum, we were able to identify specific peaks corresponding to crystalline zinc within the ZnO-NPs that were synthesized through biological means. The XRD analysis of these biosynthesized ZnO-NPs exhibited characteristic diffraction patterns. Notably, the peaks located at 2θ values of 31.6˚, 34.46˚, 36.26˚, 47.5˚, 55.54˚, 62.5˚, 65˚, and 72.4˚ were matched to the (100), (002), (101), (102), (110), (103), (200), and (004) crystallographic planes of ZnO-NPs, respectively. There were no additional diffraction peaks detected from other phases, signifying the high phase purity of the ZnO-NPs powder, as depicted in Fig. 1D. It is worth noting that the diffraction patterns observed in this study closely resembled those reported by Elbahnasawy, et al. [59]. The distribution of these particles in their suspension and the average size of the biosynthesized ZnO-NPs were ascertained using DLS analysis. ZnO-NPs were found to vary in size from 20 to 150 nm, with an average size of 17 nm and a PDI of 0.475 as shown in Fig. 1E. Because DLS analysis assesses the hydrodynamic radius, the particle size determined by DLS measurements is larger than the results from TEM [4]. The PDI value of 0.475 refers to the variation in nanoparticle size which may be attributed to the presence of some aggregation that appeared in the TEM graph.

### Preparation of stable ZnO-NPs nanofluids

**Fig. 2:**
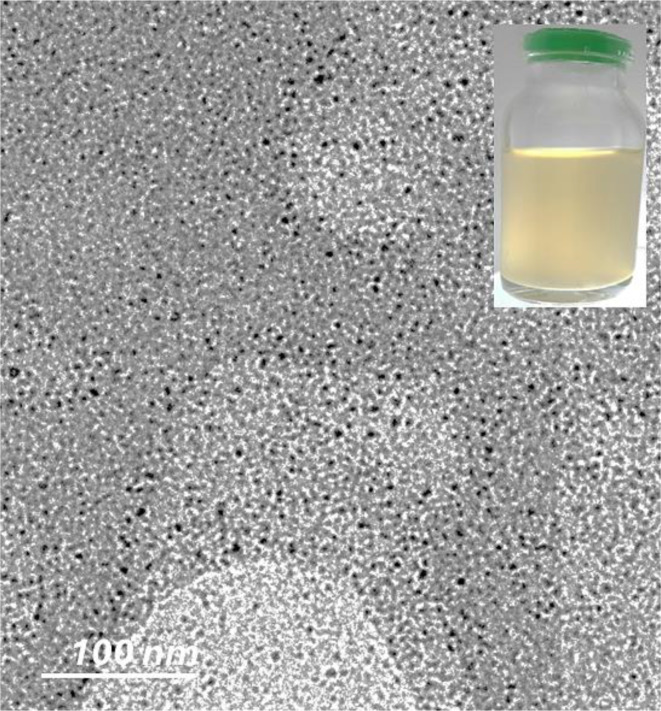
ZnO-NPs nanofluid formation was observed using HR-TEM and visual means

The most employed technique for manufacturing stable nanofluids for various research purposes involves a two-step process. Initially, dried particulate nanoparticles are generated using the previously described method. This dry material of ZnO-NPs is then mixed with a variety of liquids to produce nanofluids. The tendency of ZnO-NPs to aggregate in aqueous suspension, which may be attributed to the weakness of capping agents present in the CFF of *Streptomyces baarnensis* MH-133, is the reason why glycerol water solution (60:40 by volume) is taken as the base fluid for the preparation of ZnO-NP nanofluids in addition to ammonium hydrogen citrate, which was used as a dispersant agent. A stable nanofluid of ZnO-NPs with uniform particle dispersion is obtained, as shown in Fig. 2. HR-TEM investigation confirms the homogeneity of the ZnO-NPs nanofluid showing no aggregation in the investigated sample. Nanofluid, a colloidal suspension of nanoparticles in a regular fluid, has a greater thermal conductivity than other fluids. One of the fundamental conditions for improved exploitation of nanofluid in heat transfer applications is its long-term stability [60, 61].

### Antibacterial activity and minimal inhibitory concentrations (MICs) of nanofluid and water suspensions of ZnO-NPs

**Fig. 3:**
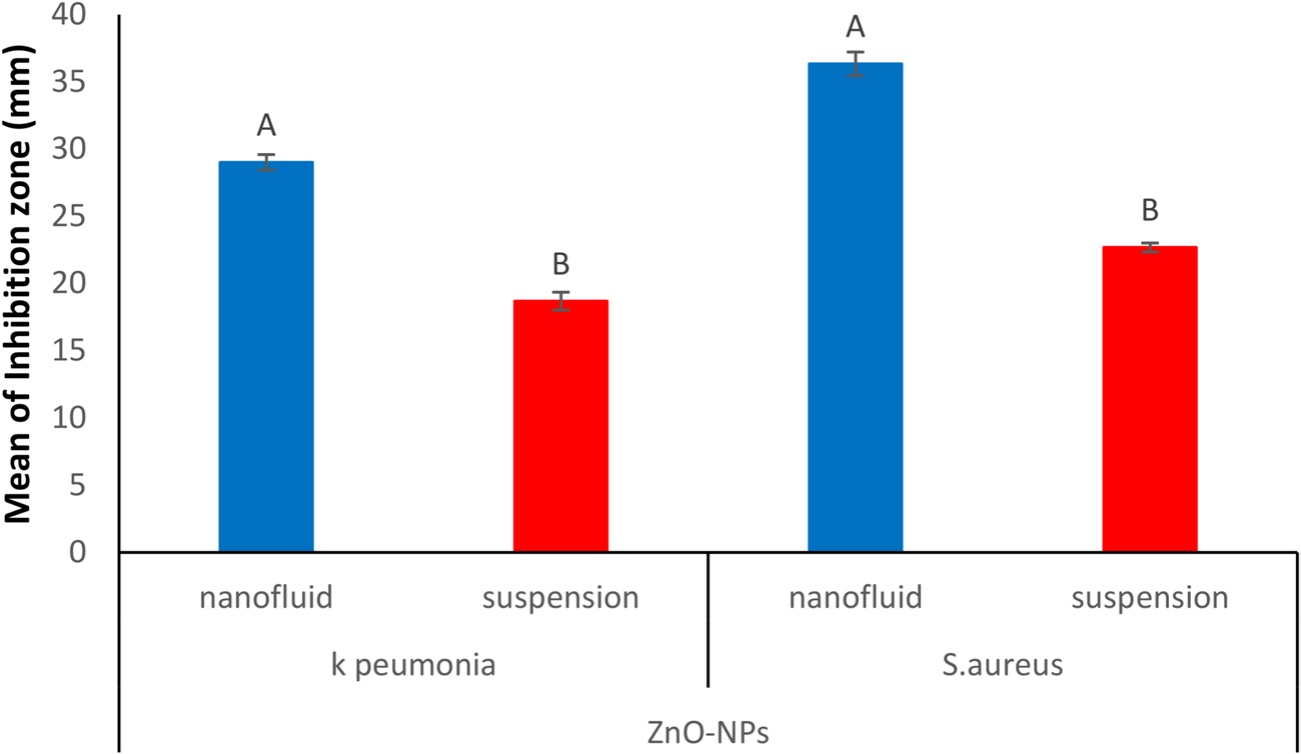
Comparison between ZnO-NPs nanofluid and water suspensions

The antibacterial activities of water suspension and nanofluid of biosynthesized ZnO-NPs were evaluated against MDR *Klebsiella pneumoniae* and MRSA, using the agar well diffusion technique. Each microorganism was treated with 100 µl of water suspension and nanofluid of ZnO-NPs at a concentration of 5 mg/ml, along with 100 µl of the nanofluid base without nanoparticles to accurately assess the activity of the suspended nanoparticles in the nanofluid. When comparing nanofluids to water suspensions of nanometals, nanofluids demonstrated a significantly greater effect against the tested microorganisms. Figure 3 shows that the nanofluid of biosynthesized ZnO-NPs exhibits high antibacterial activity, with inhibition zones of 29 ± 0.577 and 36 ± 0.882 against MDR *Klebsiella pneumonia* and MRSA, compared with ZnO-NPs water suspension which exhibited inhibition zones of 18 ± 0.667 and 22 ± 0.333 respectively. Antibacterial activity is usually dose-dependent, so it is valuable to find out the MIC values of ZnO-NPs. The results obtained from MICs showed effective inhibition of all isolates at varied concentrations of ZnO-NPs nanofluids. The MIC values of ZnO-NPs ranged from 125 µg/ml in the case of* Enterobacter cloacae*, *Acinetobacter baumanii*, *Pseudomonas aeruginosa*, and *Enterococcus faecalis* to 31.25 µg/ml in the case of *Bacillus subtilis* (ATCC-6633) and *Salmonella typhi* (ATCC-6539). The MIC values of *Escherichia coli, Klebsiella pneumonia*, and *Staphylococcus aureus* were 62.5 µg/ml, as shown in Table 1. On the other hand, the results generally reflect the resistance of the tested MDR strains if compared with tested ATCC-coded strains, where the MDR strains exhibited a higher MIC value than that of ATCC strains. The antimicrobial activity of ZnO-NPs was reported by Iqbal et al. [62]. and the MIC value was 37.5 µg/ml against *Staphylococcus aureus* ATCC 25,923, *Bacillus subtilis* ATCC 6633, *Klebsiella pneumonia* ATCC 4617, *E. coli* ATCC 15,224. According to studies on the occurrence of antibacterial activity on Zn-ONPs, Ohira et al. [63], Padmavathy and Vijayaraghavan [64]. and Jalal et al., [65], found that one of the main chemical species causing the antibacterial action was H_2_O_2_ production from the surface of ZnO nanofluid. The following explanation explains how highly reactive species like OH^−^, H_2_O_2_, and O2^2−^ are produced. Electron-hole pairs (e^−^h^+^) can be produced in ZnO with flaws because they can be triggered by both UV and visible light. ZnO nanofluid’s H_2_O molecules were divided into OH^−^ and H^+^ by the holes. Superoxide radical anions (radical dot O^− 2^) are created when oxygen molecules are dissolved in water. These radicals then combine with H^+^ to create (HO_2_ radical), which then collide with electrons to create hydrogen peroxide anions (HO_2_^−^). They then combine with hydrogen ions to form H_2_O_2_ molecules. The bacteria can be killed by the produced H_2_O_2_ by penetrating the cell membrane [64]. The hydroxyl radicals and superoxide cannot enter the cell membrane because they are negatively charged particles, thus they must stay in contact with the bacteria’s outer surface. H_2_O_2_, on the other hand, can enter the cell [65]. It is conceivable to argue that nanofluid concentrations are comparable to H_2_O_2_ concentrations. According to the earlier findings by Yamamoto [66], the amount of H_2_O_2_ produced on the ZnO surface should rise in direct proportion to an increase in nanoparticle concentration and time. The increase in H_2_O_2_ concentration produced from the ZnO surface is thought to be the cause of the antibacterial activity increasing with time and ZnO nanofluid concentration.

**Table 1:** MIC values of the biosynthesized ZnO-NPs nanofluid

No	Bacterial strains	MIC of nanofluid ZnO-NPs (µg/ml)
1	*Enterobacter cloacae*	125
2	*Acinetobacter baumanii*	125
3	*Escherichia coli*	62.5
4	*Klebsiella pneumonia*	62.5
5	*Pseudomonas aeruginosa*	125
6	*Enterococcus faecalis*	125
7	*Staphylococcus aureus*	62.5
8	*Bacillus subtilis-*ATCC-6633	31.25
9	*Salmonella typhi-*ATCC-6539	31.25

### Evaluation of combination interaction of the purified compound (Ka) and ZnO-NPs using checkerboard microdilution assay

To improve the activity of ZnO-NPs and Ka as well as to reduce their dose, different combinations of ZnO-NPs and Ka were made according to the checkerboard microdilution method. The antibacterial activity of these combinations was performed using MRSA and *Klebsiella pneumonia* as test strains. The standard checkerboard approach is a generic process for identifying synergy in which two drugs are evaluated at successive dilutions and in combinations to determine the concentration of each component that has a minimal synergistic impact with the other. The interaction can be calculated algebraically using the fractional inhibitory concentrations index (FICi). FICi is an indicator of the interaction degree between ZnO-NPs along with Ka against *Klebsiella pneumonia* and MRSA *.* When the Ka and ZnO-NPs were combined in a checkerboard matrix, 19 treatments caused inhibition of *Klebsiella pneumonia* in this assay. Eleven of them resulted from FICi ranging from (1.03 to 2) which means indifferent. Another five treatments resulted from FICi ranging from (0.625 to 1) which means additive. The results also show FICi of 0.5 meaning synergy Table 2. In other words, when Ka-ZnO-NPs combinations were tested against methicillin-resistant *S. aureus* (MRSA), it was produced 23 treatments which varied in their interaction degree. Eleven of them showed indifferent interaction, eight exhibited additive interaction but only two of them showed synergy with FICi of 0.5 and 0.375 Table 3. From the data in Tables 2 and 3, it was concluded that ZnO-NPs and Ka were powered by each other resulting in a decrease in the MIC value for both in comparison with each one of them alone. The MIC of each ingredient in the combinations that exhibited only synergy was reduced to one-fourth only in all checkerboard assays that tested against *K. pneumonia* and MRSA with the only exception in the case of checkerboard matrix of Ka-ZnO-NPs combination against MRSA where the MIC value of ZnO-NPs was decreased to become one eighth only while MIC of Ka decreased only to one fourth in the same combination.

**Table 2:** Checkerboard results of Ka- ZnO-NPs combinations against *K. pneumonia*

No.	ka/ZnO-NPs Ratio	Ka + ZnO-NPs (µg/ µg)/ml	FIC ka	FIC ZnO-NPs	FICi	Interpretation
1	MIC ⁄ MIC	300 + 62.5	1	1	2	indifferent
2	MIC ⁄ 1/2	300 + 31.25	1	0.5	1.5	indifferent
3	MIC ⁄ 1/4	300 + 15.6	1	0.25	1.25	indifferent
4	MIC ⁄ 1/8	300 + 7.8	1	0.125	1.125	indifferent
5	MIC ⁄ 1/16	300 + 3.9	1	0.0625	1.0625	indifferent
6	MIC ⁄ 1/32	300 + 1.95	1	0.03125	1.03125	indifferent
7	MIC ⁄ 0	300 + 0	1	0	1	-------
8	1/2⁄ MIC	150 + 62.5	0.5	1	1.5	indifferent
9	1/2 ⁄ 1/2	150 + 31.25	0.5	0.5	1	additive
10	1/2⁄ 1/4	150 + 15.6	0.5	0.25	0.75	additive
11	1/2 ⁄ 1/8	150 + 7.8	0.5	0.125	0.625	additive
12	1/4/MIC	75 + 62.5	0.25	1	1.25	indifferent
13	1/4/1/2	75 + 31.25	0.25	0.5	0.75	additive
14	1/4/1/4	75 + 15.6	0.25	0.25	0.5	synergistic
15	1/8/MIC	37.5 + 62.5	0.125	1	1.125	indifferent
16	1/8/1/2	37.5 + 31.25	0.125	0.5	0.625	additive
17	1/16/MIC	18.75 + 62.5	0.0624	1	1.0624	indifferent
18	1/32/MIC	9.375 + 62.5	0.03124	1	1.03124	indifferent
19	0/MIC	0 + 62.5	0	1	1	-------

**Table 3:** Checkerboard results of Ka- ZnO-NPs combination against MRSA

No.	ka/ZnO-NPs Ratio	ka/ZnO-NPs (µg/ µg)/ml	FIC ka		FIC ZnO-NPs	FICi	Interpretation
1	MIC ⁄ MIC	300 + 62.5		1	1	2	indifferent
2	MIC ⁄ 1/2	300 + 31.25		1	0.5	1.5	indifferent
3	MIC ⁄ 1/4	300 + 15.6		1	0.25	1.25	indifferent
4	MIC ⁄ 1/8	300 + 7.8		1	0.125	1.125	indifferent
5	MIC ⁄ 1/16	300 + 3.9		1	0.0625	1.0625	indifferent
6	MIC ⁄ 1/32	300 + 1.95		1	0.03125	1.03125	indifferent
7	MIC ⁄ 0	300 + 0		1	0	1	--------
8	1/2⁄ MIC	150 + 62.5		0.5	1	1.5	indifferent
9	1/2 ⁄ 1/2	150 + 31.25		0.5	0.5	1	additive
10	1/2⁄ 1/4	150 + 15.6		0.5	0.25	0.75	additive
11	1/2 ⁄ 1/8	150 + 7.8		0.5	0.125	0.625	additive
12	1/2⁄ 1/16	150 + 3.9		0.5	0.0625	0.5625	additive
13	1/2⁄ 1/32	150 + 1.95		0.5	0.03125	0.53125	additive
14	1/4/MIC	75 + 62.5		0.25	1	1.25	indifferent
15	1/4/1/2	75 + 31.25		0.25	0.5	0.75	additive
16	1/4/1/4	75 + 15.6		0.25	0.25	0.5	synergism
17	1/4/1/8	75 + 7.8		0.25	0.125	0.375	synergism
18	1/8/MIC	37.5 + 62.5		0.125	1	1.125	indifferent
19	1/8/1/2	37.5 + 31.25		0.125	0.5	0.625	additive
20	1/16/MIC	18.75 + 62.5		0.0624	1	1.0624	indifferent
21	1/16/1/2	18.75 + 31.25		0.0624	0.5	0.5624	additive
22	1/32/MIC	9.375 + 62.5		0.0312	1	1.03124	indifferent
23	0/MIC	0 + 62.5		0	1	1	---------

A few years ago, monotherapy was the most common way to treat infectious diseases. But now, there is enough proof to show that combination antimicrobials or multitherapy work better than single-drug treatments [67]. The checkerboard broth microdilution test is a common way to find out how mixed antimicrobials work. The results are used to show whether the antimicrobials are indifferent, additive, synergistic, or antagonistic effects [68]. According to the data obtained from the combination of the Ka and ZnO-NPs test, it is obvious that the checkerboard assay identified the concentration of each agent in the combination when the FIC index ≤ 0.5. This synergistic combination of Ka and ZnO-NPs reduces the dose of both agents (to at least one-fourth of the corresponding MIC), potentially reducing toxicity while increasing antibacterial capabilities. When mixed with antibiotics like β-lactams, cephalosporins, and aminoglycosides, ZnO NPs have also been shown to improve drugs’ ability to kill different harmful bacteria. Bhande et al. [69], found that ZnO nanoparticles worked better against bacteria when mixed with the β-lactam drugs cefotaxime, ampicillin, ceftriaxone, and cefepime, in that order, against *E. coli, K. pneumoniae, S. paucimobilis, and P. aeruginosa.* What they found was that ZnO nanoparticles made β-lactam drugs kill bacteria between 50% and 85% more effectively than these regular antibiotics used alone. Abo-Shama et al. [27], also found that the antibiotics azithromycin, oxacillin, cefotaxime, cefuroxime, fosfomycin, and oxytetracycline worked better together against *E. coli* and *S. aureus* than when used alone or with ZnO-NPs. The impact of these combinations may result from the oxidative stress induced by nanoparticles on the cell surface, which led to a rise in the permeability of the cell membrane, thus causing the enhancement of Ka entry and increasing its concentrations at the site of antibiotic-microbe contact [70].

### Development and characterization of nanoemulsion loaded with ka and ZnO-NPs combination

**Fig. 4:**
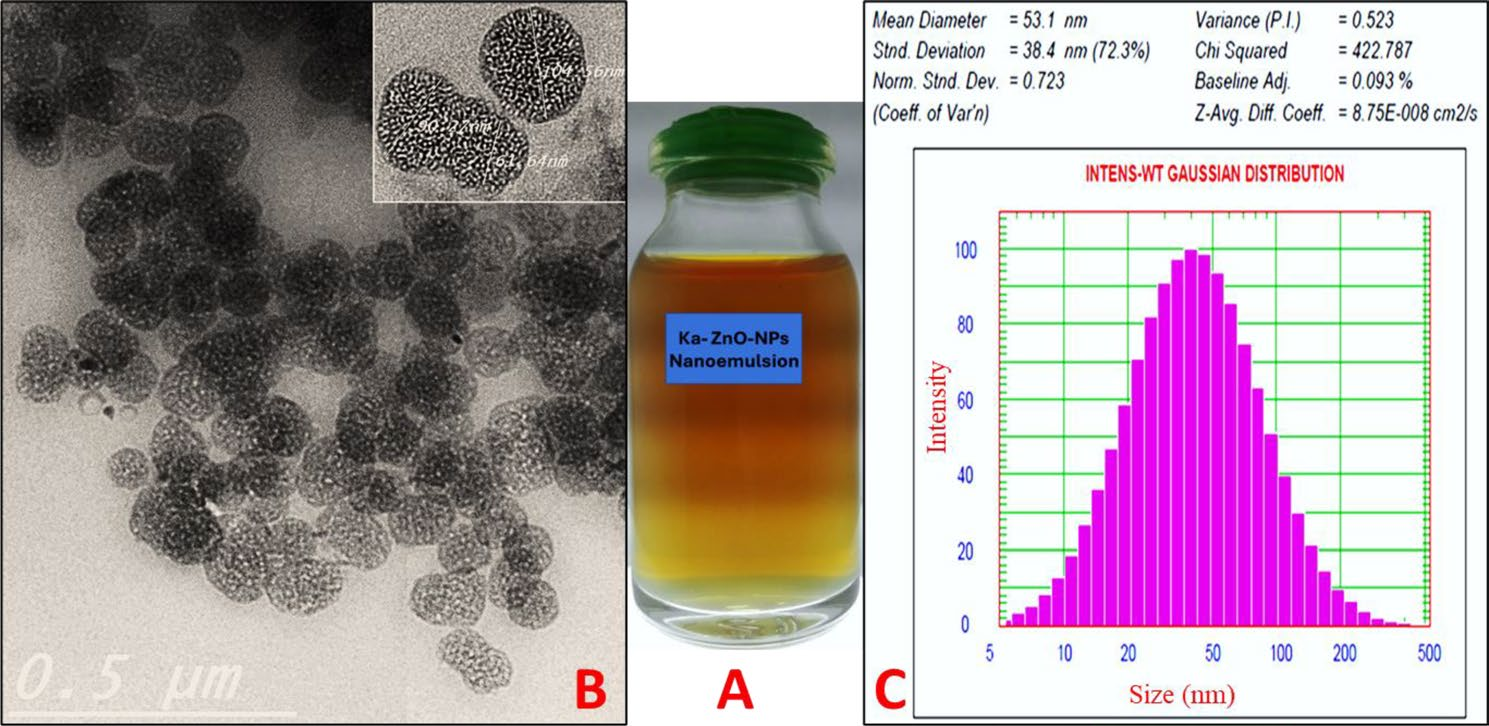
Characterization of nanoemulsion loaded with ka and ZnO-NPs combination. (**A**) visual observation of nanoemulsion loaded with ka and ZnO-NPs combination, (**B**) Transmission electron micrographs, (**C**) Particle size distribution

The prepared formula (after ka and ZnO-NPs loading) was observed after 24 h for the detection of any turbidity, creaming, precipitation, or phase separation. This formula appeared as a clear nanoemulsion without phase separation Fig. 4A. The High-resolution transmission electron microscopy (HRTEM) analysis revealed a positive image in which nanoemulsion oil droplets appeared as dark spheres with bright surroundings. The micrograph exhibits, the droplet size of nanoemulsion appeared as a regular spherical shape with a granulated core without aggregations and the size of these droplets was less than 200 nm Fig. 4B. Dynamic Light Scattering analysis was done to determine the average diameter of the nanoemulsion droplets loaded with Ka-ZnO-NPs combination. The results indicated that the nanoemulsion droplets were found in the nano range with an average size of 53.1 nm and PI value of 0.523 meaning an acceptable distribution of nanoemulsion droplets Fig. 4C.

Over 40% of newly identified chemical compounds in the pharmaceutical industry have a high level of insolubility in water. For the medication to be effectively absorbed, it must be in a soluble state at the location of absorption [71]. Various formulation techniques have been proposed to address these challenges, including lipid-based drug delivery systems such as nanoemulsions, which are well-regarded for their potential as an alternative strategy for delivering hydrophobic drugs. A nanoemulsion is a thermodynamically stable solution comprising an oil, surfactant, cosurfactant (solubilizer), and drug combinations that spontaneously form an oil-in-water (o/w) nanoemulsion when mixed with water under stirring [72]. In the present study olive oil, tween-80, and glycerol were selected for the preparation of nanoemulsion at a ratio of 45% of S_mix_ (2:1), 7.5% of olive oil, and 47.5% water. Olive oil which was used to dissolve the content of ka is known to be the oil that has the highest content of fatty acids, namely palmitic acid, oleic acid, and linoleic acid which act as an enhancer for the bioavailability of hydrophobic drug substances [73]. Surfactants lower surface tension and promote hydrophobic medication solubility in aqueous solutions. They may also be employed to keep drug suspension stable. Non-ionic surfactants, such as Tween 80, were employed since they are well-known for their non-irritant properties and are usually considered safe and biocompatible [74]. The addition of glycerol as a co-surfactant reduces the bending stress of the interface and enables the interfacial layer considerable flexibility to take up the varied curvatures necessary to produce a nanoemulsion throughout a broad variety of compositions [75]. TEM micrograph confirmed the sphericity of the nanoemulsion droplets which loaded with Ka-ZnO-NPs combination, due to the particles showing spherical with granulated core and without aggregation, also seem symmetrical size. These results confirmed that the droplets were in the nano-size range (less than 200 nm). These results are those reported by many researchers [76, 77]. Particle size and particle size distribution expressed as polydispersity index (PI) are two important parameters since they will affect the saturation solubility and stability of nanoemulsion [78]. The data obtained from the TEM micrograph of nanoemulsion was confirmed with DLS analysis which indicated that nanoemulsion droplets loaded with Ka-ZnO-NPs showed a particle size average of 53.1 nm with a polydispersity index (PI) of 0.52 confirming the formation of nanoemulsion.

### Comparative studies between formulated combinations (nanoemulsion) and non-formulated combinations

#### Time kill assay

**Fig. 5:**
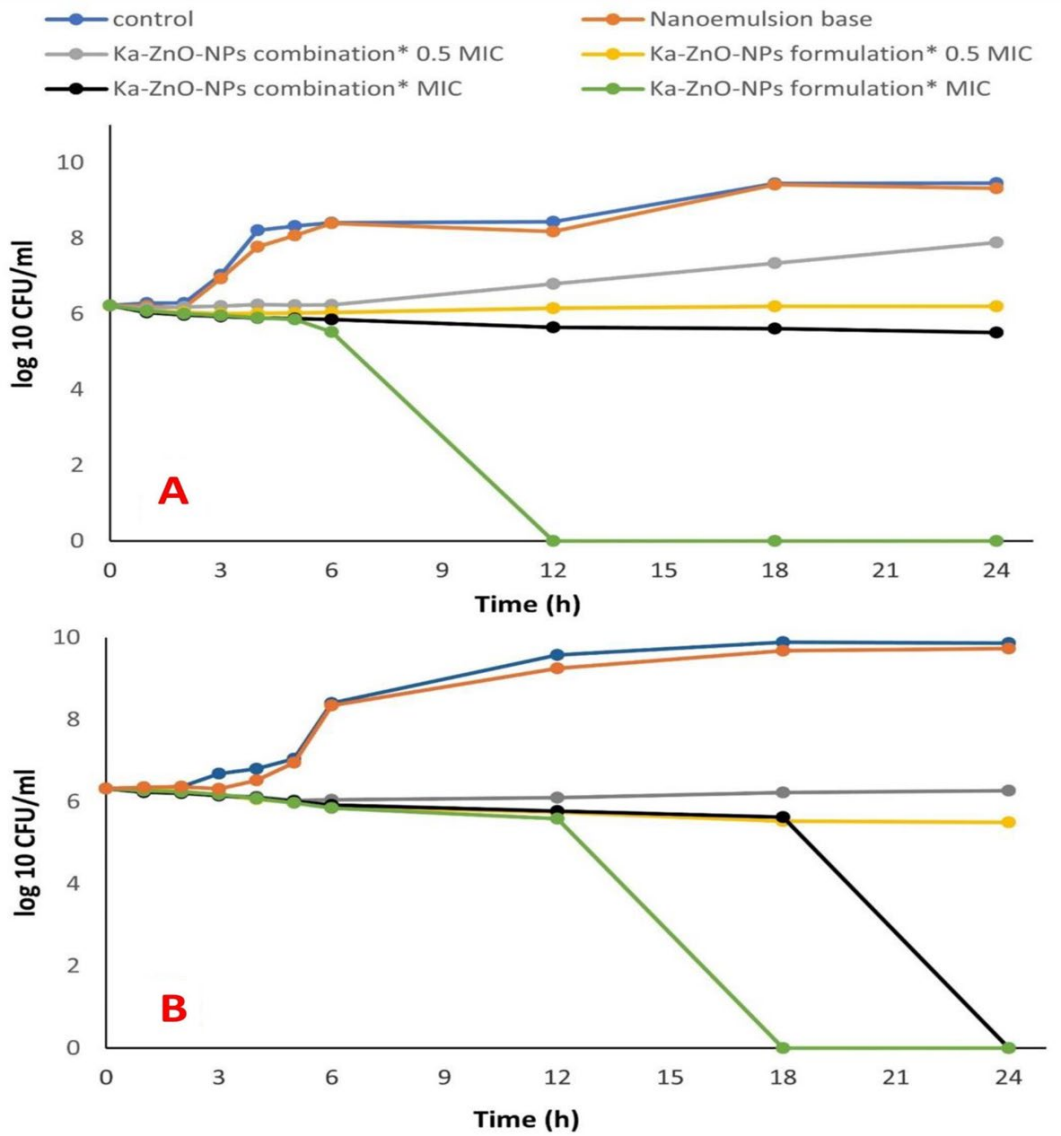
Time kill assay. (**A**) MRSA , (**B**) *K. pneumonia* treated with different forms of Ka-ZnO-NPs combinations

The killing kinetic assay was utilized to determine the minimum time required for both the formulated and unformulated combinations to achieve an inhibitory or bactericidal effect on post-treatment bacterial viability. This experiment used the half MIC value of the Ka-ZnO-NPs combination, obtained from the checkerboard assay, as well as the MIC concentration against MRSA and *K. pneumoniae*. Data transformation from absolute CFU/ml to log_10_ values allowed for the calculation of the increase or decrease in log_10_ (CFU/ml) of the bacterial strains after treatment. Consequently, the inhibitory or lethal effect of these combination forms was easily determined by subtracting the log_10_ (CFU/ml) value at any given time from the log_10_ (CFU/ml) of the initial inoculum. In this assay, MRSA was treated with the Ka-ZnO-NPs combination at two concentrations (0.5 Χ MIC = 37.5 + 3.9 and 1 Χ MIC = 75 + 7.8 (µg/µg)/ml of Ka and ZnO-NPs, respectively), and each concentration was tested in two forms (nanoemulsion and non-formulated form). In Fig. 5A, the growth of treated MRSA was varied according to the form and concentration of these treatments. The greater effect was obvious with the high concentration, especially in the nanoemulsion form. As it is prevalent, the Ka-ZnO-NPs combination at only 0.5 MIC concentration in non-formulated form prevented the log_10_ CFU /ml of initial inoculum from increasing for six hours after that, it was increased to reach 1.6679 log_10_ CFU /ml above the log_10_ CFU /ml of initial inoculum after 24 h of incubation. The nanoemulsion form at the same concentration suppressed the log_10_ CFU /ml of initial inoculum from the first hour of contact causing reduction in their number; the maximum reduction was observed at the third hour of incubation (-0.2179 log_10_ CFU/ml), then the viable cells were increased gradually in the next time to minimize this reduction to -0.0193 log_10_ CFU/ml under the log_10_ CFU/ml of initial inoculum after 24 h. The increasing of Ka-ZnO-NPs concentration from 0.5 Χ MIC to 1Χ MIC value inhibited the growth of MRSA either by formulated or non-formulated form. The results demonstrated that the nanoemulsion of Ka-ZnO-NPs combination increased the inhibitory effect to become lethal where the log_10_ CFU number of MRSA was reduced from the first hour of contact until the complete eradication (-6.2279 log_10_) was done after 12 h of incubation and extended to the end of the experiment. In other words, the non-formulated form of Ka-ZnO-NPs combination at 1 Χ MIC value also reduced the log_10_ CFU number of MRSA during different time intervals and the maximum reduction was observed after 24 h of incubation which reached − 0.7138 log_10_ under the log_10_ CFU /ml of initial inoculum.

*K. pneumonia* was subjected to Ka-ZnO-NPs combination at 0.5 MIC value (37.5 + 7.8 as well as 1 Χ MIC concentration (75 + 15.6) µg/ µg/ml of Ka and ZnO-NPs respectively. The inhibitory or lethal effect of Ka-ZnO-NPs combination against *K. pneumonia* was different than the previous cases where all formulated and non-formulated treatments caused a reduction of log_10_ CFU number under the limit of CFU/ml of initial inoculum Fig. 5B, Ka-ZnO-NPs combination at 0.5 MIC concentration in non-formulated form suppressed the log_10_ CFU/ml from the first hour of incubation until reached at the maximum reduction (-0.2896 log_10_) after 5 h of incubation. Then the cells of *K. pneumonia* were increased again over time, but the growth remained under the level of initial inoculum until the end of the experiment (-0.0536 log_10_) under the log_10_ CFU/ml of initial inoculum. The nanoemulsion form of Ka-ZnO-NPs combination at 0.5 MIC concentration also decreased the log_10_ CFU number from the first hour of contact and its effect continued to decrease the log_10_ CFU/ml number until it reached − 0.8264 log_10_ under the log_10_ CFU/ml of initial inoculum after 24 h of incubation. As is familiar from previous cases, the increase of concentration from 0.5 Χ MIC to 1 Χ MIC caused more reduction in the log_10_ CFU/ml number; this reduction was increased by time causing complete eradication of log_10_ CFU/ml (-6.327 log_10_) of *K. pneumonia* which was treated with Ka-ZnO-NPs combination at 1 Χ MIC value after 24 h of incubation. On the other side, the nanoemulsion form of this combination also reduced the log_10_ CFU/ml number but in a manner more rapid than that caused by the non-formulated form where it caused complete eradication (-6.327 log_10_) only after 18 h of incubation. Finally, it’s important to refer to the non-inhibitory effect of empty nanoemulsion against MRSA and *K. pneumonia* at all time intervals where the growth curve resembles that of control.

Time-kill studies provide insights into not only the bactericidal or bacteriostatic properties of formulated and unformulated combinations but also the duration required for these formulations to eliminate the microorganism [79]. The kinetics of antibacterial activity of nanoemulsion was evaluated against MRSA and *K. pneumonia* according to guidelines of CLSI, [46]. According to Lorian [47], an agent is considered bactericidal for a specific pathogen if it reduces the bacterial count by 3 log_10_ CFU/ml within 24 h of incubation in liquid media. Bacteriostatic, on the other hand, is defined as a decrease of less than 3 log_10_ CFU/ml relative to the initial inoculum. The results indicate that the combinations of Ka-ZnO-NPs without formulation at MIC values showed a bactericidal effect against *K. pneumonia* after 24 h but did not show the same effect against MRSA. Surprisingly, our developed nanoemulsion loaded with the previously mentioned combinations at MIC value displayed a marked increase in antibacterial activity over free combinations where nanoemulsion showed bactericidal effect at MIC concentration of Ka-ZnO-NPs combination after 12 and 18 h of incubation against MRSA and *K. pneumonia* respectively. At half MIC value, nanoemulsion increased the activity of the combinations to cause bacteriostatic effect on both MRSA and *K. pneumonia* after 24 h of incubation while the half MIC value of free combination exhibited bacteriostatic effect for a time not exceeding 6 h then the cells of the tested bacteria regrown again to increase the log_10_ cfu/ml over the initial level. Wang et al. [80] reported that based on the checkerboard MIC study, fisetin and polymyxin E worked well together against MCR-1-positive *S. typhimurium* HYM2 and *E. coli* ZJ487, with all FICI values being less than 0.5. This mixture was also put together in the form of nanoemulsion. The results showed that the fisetin nanoemulsion was a great adjuvant that made polymyxin E much more effective at fighting the infection of MCR-1-positive *S. typhimurium* HYM2 in both mouse and chick hosts. This considerable difference between the effect of formulated and non-formulated combinations may be due to the availability of nanoemulsion components with considerable amounts at the site of action due to their ability to penetrate the cell membrane [81, 82].

#### Cytotoxicity study

**Fig. 6:**
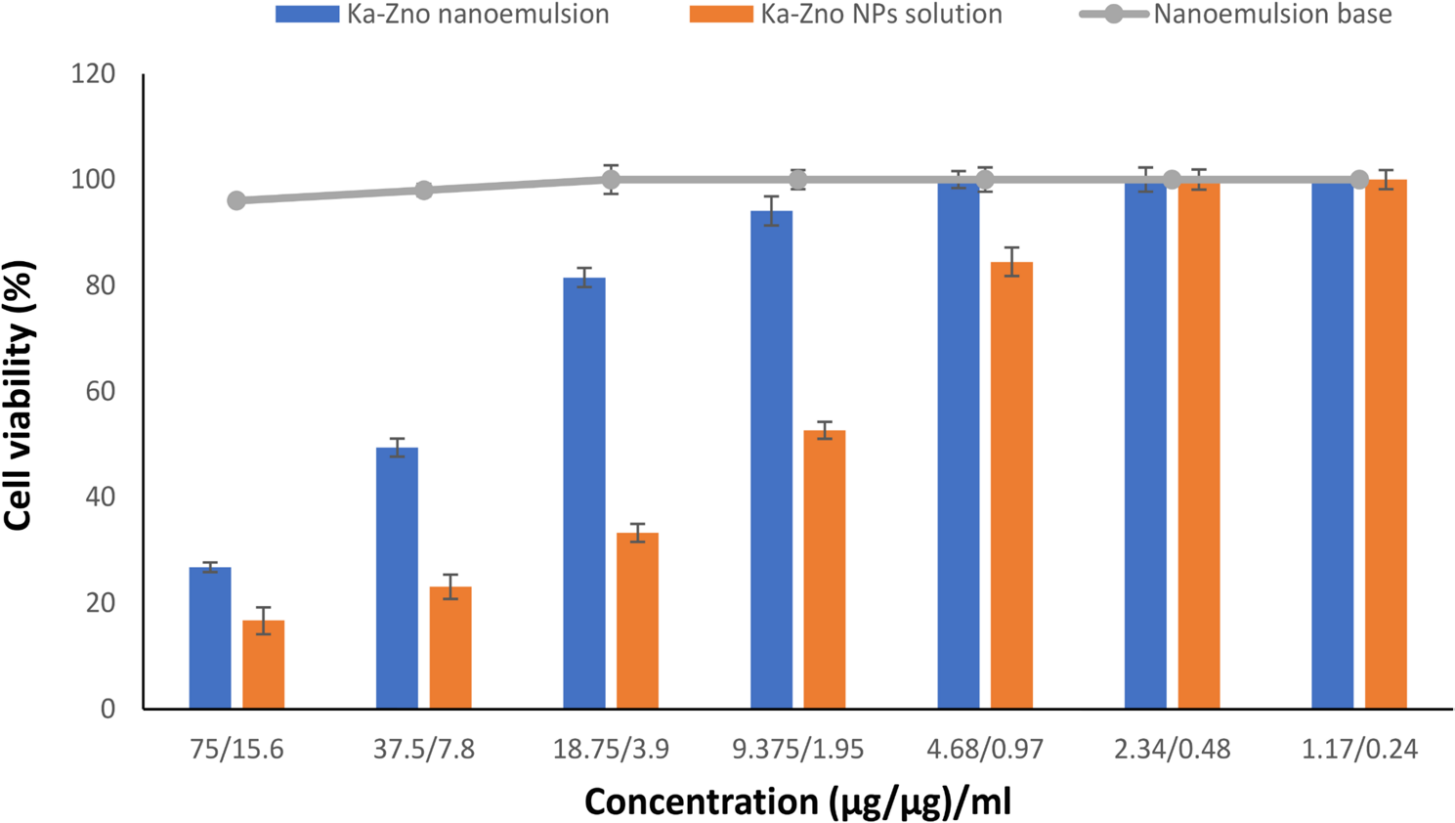
Viability of Vero cells treated with different forms of Ka-ZnO-NPs combinations

A cytotoxicity study was performed to evaluate the reduction of Vero cell growth caused by different forms of Ka-ZnO-NPs combination at different concentrations in comparison with control cells (untreated). Similarly, Vero cell viability was affected with Ka-ZnO-NPs combination in concentration-dependent behavior even if treated with nanoemulsion form or non-formulated form. The results that are represented graphically in Fig. 6, refer to the fact that the formulation of this combination in nanoemulsion form decreased the cytotoxicity if compared with the same combination in non-formulated form. It is clear to note that, the IC_50_ caused by nanoemulsion was higher than IC_50_ caused by the same combination without formulation as represented in Table 4.

**Table 4:** Viability of Vero cells treated with different forms of Ka-ZnO-NPs combinations

Treatment	IC_50_
Ka-ZnO-NPs nanoemulsion	22.94 + 4.77
Ka-ZnO-NPs solution	8.17 + 1.69
Empty nanoemulsion	36.57 + 8.17

To assess the biocompatibility of the nanoemulsion formulation, an in vitro cytotoxicity study was conducted on Vero cells. Viability testing measures the ability of cells or tissues to sustain or recover metabolic activity after exposure to various concentrations of specific chemicals, while toxicity indicates the extent to which a substance can cause harmful or lethal effects to cells [83]. The results demonstrated that the empty nanoemulsion (nanoemulsion base) has very low toxicity and can serve as a drug delivery system, reducing the toxicity of the Ka-ZnO-NPs combinations. Specifically, Vero cells treated with the non-formulated Ka-ZnO-NPs combination (absolute solution) exhibited an IC_50_ of 8.17 + 1.69 (µg/µg)/ml, whereas the IC_50_ for the same combination in the form of a nanoemulsion was achieved at a concentration of 22.94 + 4.77 (µg/µg)/ml. Winter et al. [84], observed that lipid nanoparticles in the form of a nanoemulsion did not cause cytotoxicity in Vero cells even after 48 h of incubation. It has also been observed that the toxicity of nanoparticles is determined by physiochemical characteristics such as particle size, shape, surface charge, composition, and formulation stability.

#### Observation of the effects nanoemulsion combinations on bacterial cells via TEM

**Fig. 7:**
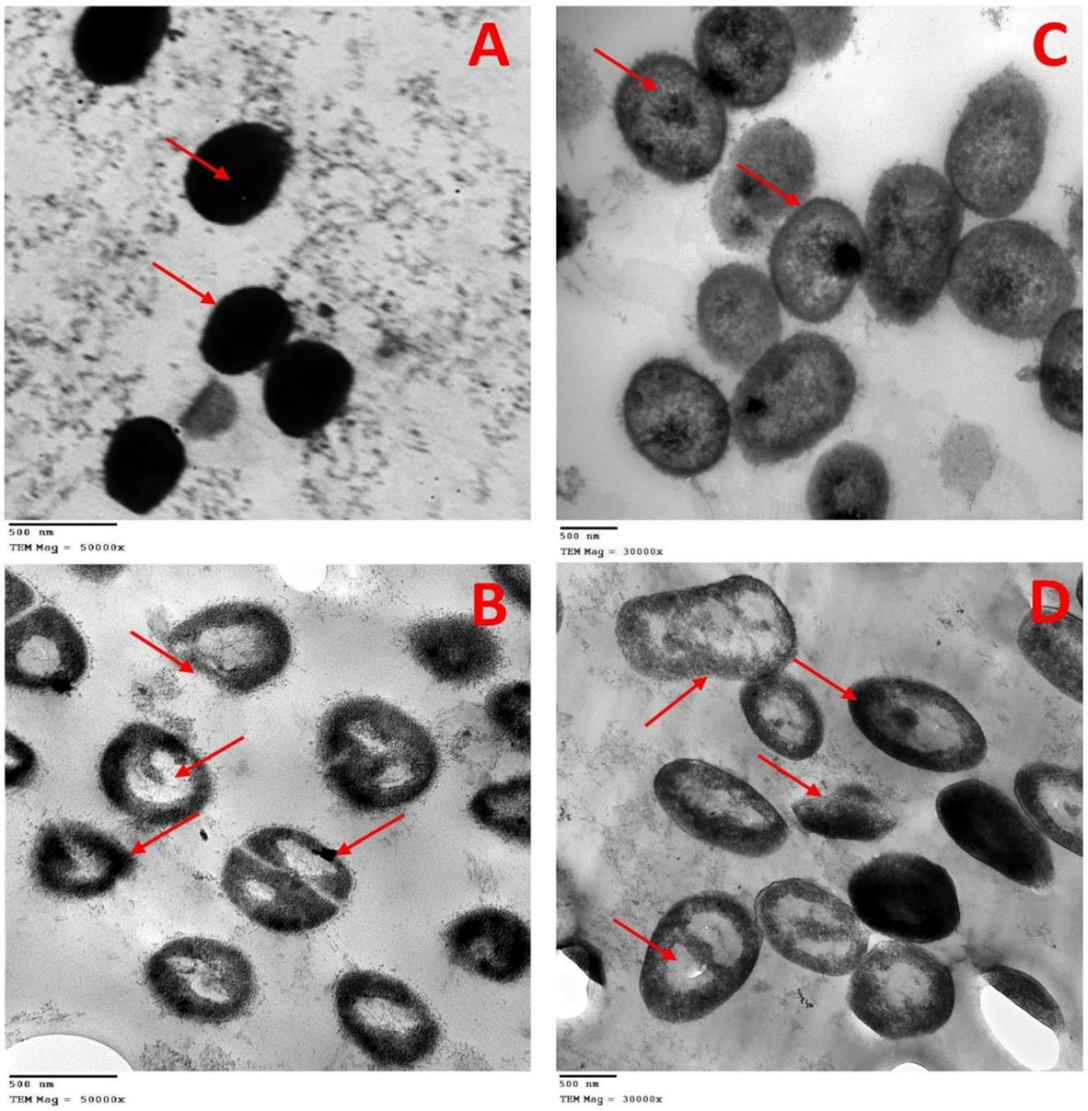
Transmission electron microscope (TEM) images which visualize the morphological changes in *S. aureus* and *K. pneumonia* upon treating with Ka-ZnO-NPs nanoemulsion at low concentration: (**A**) untreated, (**B**) treated cells of *S. aureus* (C) untreated and (D) treated cells of *K. pneumonia*

Based on the results of time-kill and cytotoxicity assays, the Ka-ZnO-NPs nanoemulsion was chosen to study its effects on MRSA and *Klebsiella pneumoniae* using transmission electron microscopy (TEM). The bacteria were treated with the Ka-ZnO-NPs nanoemulsion at a low concentration (one-fourth of the MIC value determined by the checkerboard assay) to observe the morphological changes in the bacterial cells post-treatment. Micrographs of untreated MRSA cells (control) showed normal spherical cells with intact cell walls and membranes, along with dense cytoplasmic areas. The cell wall appeared with a moderately rough cell surface Fig. 7A. The treated bacterial cells of MRSA seemed to have abnormal morphological changes, although the cell size was like the untreated cells the cells appeared with a high degree of deformation, and most of the cells appeared with rough and breached cell walls. The density of cytoplasm is reduced until a vacuole-like form (vacuolation) is observed in Fig. 7B. In contrast, the micrographs of the untreated cells of *Klebsiella pneumoniae*, another bacterial model used in this work, exhibited a characteristic morphology of short rods with intact cell walls, outer and cytoplasmic membranes, and cytoplasmic content including only a few dense patches. The cell surface exhibited the usual features of native cells, including a smooth and undamaged appearance, with some visible filaments surrounding the cells Fig. 7C. The morphological abnormalities that were seen after treatment of *Klebsiella pneumoniae* cells with nanoemulsion of Ka-ZnO-NPs were also varied. Some irregular forms of cells were seen with ruptured cell walls and membranes. The reduction in cytoplasm density has also appeared as that found in MRSA resulting in the appearance of vacuolation in most of the cells Fig. 7D.

From the micrographs, it is evident that the untreated bacterial cells of MRSA and *K. pneumonia* remained intact without any changes in their shape, while the cells treated with nanoemulsion exhibited disruption and disintegration of the bacterial cell wall, leading to a noticeable alteration in their morphological structure. Certain regions of the membrane exhibit visible damage and there is evident leaking of intracellular components. The bacterial cell membrane plays a crucial role in important processes such as regulating osmotic pressure, facilitating transport, and cross-linking peptidoglycan. The preservation of the bacterial membrane’s integrity is vital for the organism’s survival since any disturbance to it might result in the death of the cell, either directly or indirectly [85]. The effectiveness of nanoemulsion in combating bacterial cells is based on its surface composition and its interaction with the cell surface. It enhanced the solubility and bioavailability of the active components and facilitated their penetration into the bacterial cell. The nanoemulsion has become critical in causing physical harm to bacteria by merging with their cell wall and membrane. This fusion allows a greater amount of loaded compound to enter the bacterial cells, facilitated by the electrostatic interaction between the cationic charge of the nanoparticles and the anionic charge on the microorganisms [86]. Ultimately, the destabilization of the lipid bilayers in the membrane and the resulting impairment of cellular permeability makes it a powerful antibacterial agent. Additionally, this mechanism may be preventing bacteria from gaining resistance [87].

## Conclusions

ZnO nanoparticles (ZnO-NPs) can be synthesized using a green synthesis technique, which aims to enhance their quality without compromising cost or safety, thereby offering new therapeutic potentials due to their unique particle size. *Streptomyces baarnensis* MH-133 was utilized in the biosynthesis of ZnO-NPs, confirming their successful production, purification, and antibacterial potential. Additionally, incorporating the water-insoluble compound 9-Ethyl-1,4,6,9,10-pentahydroxy-7,8,9,10-tetrahydrotetracene-5,12-dione (Ka) into ZnO-NPs demonstrates a strategic approach to enhancing their therapeutic efficacy. Novel formulation procedures were used to create a nanoemulsion that improves the solubility and bioavailability of the Ka-ZnO-NPs combination, increasing its potency against clinically relevant infections. Post-treatment viability and cytotoxicity assessments against methicillin-resistant *Staphylococcus aureus* (MRSA) and *Klebsiella pneumoniae* provide valuable insights into the safety and effectiveness of the Ka-ZnO-NPs combination. The killing kinetic test highlights the temporal dynamics of bacterial suppression, while cytotoxicity studies offer critical information on the biocompatibility of the nanoemulsion formulation. These findings suggest that ZnO-NPs nanoemulsions could be pivotal in combating pathogenic bacteria in various biotechnological applications. Future research should focus on developing and optimizing novel ZnO-NPs and Ka formulations, such as nanoemulsions, to enhance stability, solubility, and bioavailability. Extensive in vivo testing is necessary to evaluate their safety, biocompatibility, pharmacokinetics, and therapeutic effectiveness. Comprehensive toxicological studies should be conducted to ensure the long-term safety of ZnO-NPs and their formulations. Furthermore, scalable and cost-effective manufacturing techniques must be developed to facilitate commercialization and industrial applications.

## Data Availability

All data generated or analyzed during this study are included in this published article.
